# First complete genome sequence of tulip mild mottle mosaic virus (*Ophiovirus tulipae*)

**DOI:** 10.1007/s00705-025-06224-6

**Published:** 2025-01-17

**Authors:** Yutaro Neriya, Kakeru Hamamoto, Tominari Kobayashi, Shunsuke Nakase, Rena Kurosawa, Tomohiro Suzuki, Hisashi Nishigawa, Toshiyuki Morikawa, Tomohide Natsuaki

**Affiliations:** 1 School of Agriculture, Utsunomiya University, 350 Mine-machi, Utsunomiya, Tochigi 321-8505 Japan; 2 Center for Bioscience Research and Education, Utsunomiya University, 350 Mine-machi, Utsunomiya, Tochigi 321-8505 Japan; 3 Toyama Prefectural Agricultural, Forestry and Fisheries Research Center, 1124-1 Yoshioka, Toyama, 939-8153 Japan; 4 Present Address: Division of Virology, Department of Infection and Immunity, Jichi Medical University School of Medicine, Shimotsuke, Tochigi 329-0498 Japan; 5 Present Address: Japan Plant Protection Association, 2-28-10 Nakazato, Kita-ku, Tokyo, 114-0015 Japan

## Abstract

Tulip mild mottle mosaic disease, caused by tulip mild mottle mosaic virus (TMMMV, species *Ophiovirus tulipae*), was first reported in Japan in 1979. TMMMV has a negative-sense ssRNA genome and is closely related to ophioviruses such as Mirafiori lettuce big vein virus (MLBVV, *Ophiovirus mirafioriense*). However, its complete nucleotide sequence has not yet been reported. Therefore, in this study, we determined the complete nucleotide sequences of all four TMMMV segments. The 5′-terminal tetranucleotide sequences were highly conserved, and the five bases at the 5′ end were identical in all segments. Nucleotide sequence comparisons of the four segments showed the highest sequence identity to those of MLBVV (70–76% for the genomic RNA sequences and 81–87% for the amino acid sequences of each ORF, except for ORF2). The amino acid sequences of the coat proteins of TMMMV and MLBVV were found to be 83.1% identical, which is below the 85% threshold for assignment to the same species, indicating that these viruses belong to different species in the same genus.

**Supplementary Information:**

The online version contains supplementary material available at 10.1007/s00705-025-06224-6.

Mild mottle mosaic symptoms were first observed on tulips (*Tulipa gesneriana* and *Tulipa* sp.) in Toyama Prefecture, Japan, in 1979 (Fig. 1A) [1]. The diseases responsible for these symptoms cause severe problems and have spread to bulb-producing areas in Japan. Mild mottle disease is caused by tulip mild mottle mosaic virus (TMMMV, species *Ophiovirus tulipae*), which is a member of the genus *Ophiovirus* in the family *Aspiviridae* (formerly *Ophioviridae*) [2, 3]. TMMMV is transmitted by the obligate parasitic soil-borne fungus *Olpidium virulentus* (synonym *Ol. brassicae*) [4, 5].

**Fig. 1:**
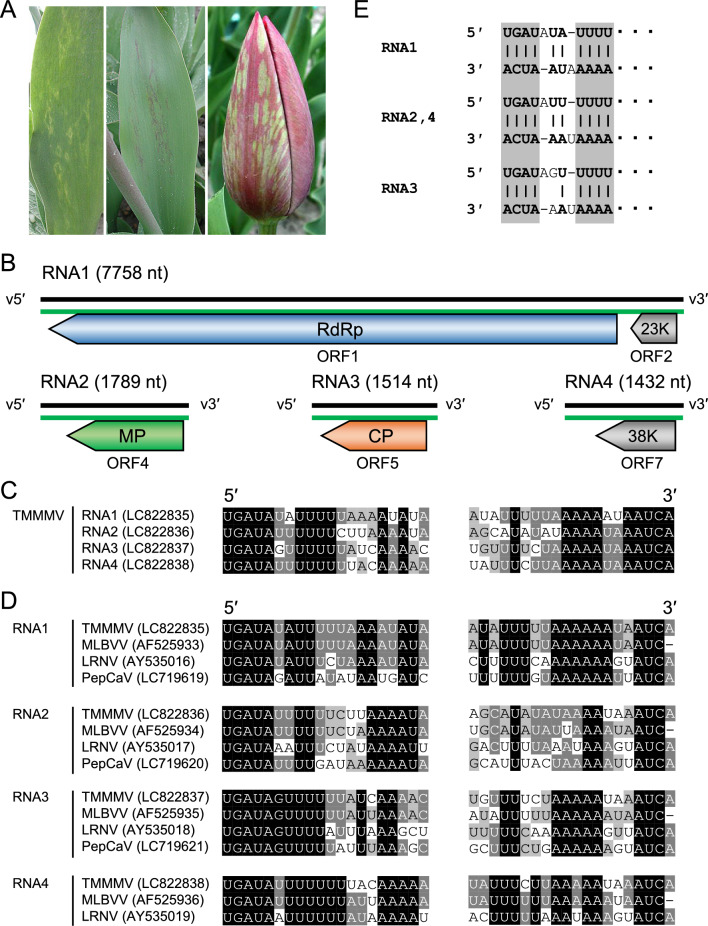
(A) Symptoms of tulip mild mottle mosaic disease. (B) Schematic diagram of the viral and complementary strands of the TMMMV genome. Black and green lines indicate viral RNA and viral complementary RNA, respectively. ORF, open reading frame; RdRp, RNA-dependent RNA polymerase; MP, movement protein; CP, coat protein; 23K and 38K, unknown proteins. Alignment of the 5′- and 3′-terminal sequences of (C) the four TMMMV segments and (D) TMMMV and other ophioviruses. DDBJ/ENA/GenBank accession numbers are shown in parentheses. Black and gray backgrounds indicate the degree of conservation, with darker shades indicating higher conservation. (E) Panhandle structures formed by the 5′ and 3′ termini of the TMMMV genome segments. The gray background indicates the region that is conserved in all segments.

TMMMV is closely related to Mirafiori lettuce big vein virus (MLBVV, species *Ophiovirus mirafioriense*). The nucleotide sequence of TMMMV RNA3, which encodes the viral coat protein (CP), has already been determined (DDBJ/ENA/GenBank accession numbers AY542958 and KY769675, from Japan and South Africa, respectively). However, the complete genome sequence of TMMMV has not yet been reported. Therefore, in this study, we determined the complete nucleotide sequence of TMMMV.

First, we extracted total RNA from a TMMMV-infected tulip (*Tulipa gesneriana*) flower from Toyama Prefecture, Japan, using an RNeasy Plant Mini Kit (QIAGEN, Hilden, Germany). To determine the genome sequence of TMMMV, degenerate primers were designed based on the sequences of MLBVV and citrus psorosis virus, which are both member of the genus *Ophiovirus* (accession numbers AY535016–9 and AY654892–4) (Supplementary Table S1). The DNA fragments obtained by reverse transcription (RT)-PCR using these degenerate primers were cloned into pGEM-T Easy Vector (Promega, USA) and then sequenced using a SupreDye Cycle Sequencing Kit v3.1 (M&S Technosystems, Osaka, Japan) and an Applied Biosystems 3500 Genetic Analyzer (Thermo Fisher Scientific, MA, USA). Nearly complete sequences were obtained, lacking only the terminal ends of RNA1 and RNA2. No DNA fragments derived from RNA4 were amplified.

Next, we performed high-throughput sequencing (HTS) analysis using a MiSeq platform (Illumina, CA, USA) and searched for sequences homologous to MLBVV among the contigs obtained by *de novo* assembly. In addition to the total RNA obtained as described above, double-stranded RNA (dsRNA) was also extracted according to Okada et al. [6]. MiSeq libraries were synthesized using the extracted total RNA or dsRNA using a KAPA Stranded RNA-Seq Library Preparation Kit (KAPA Biosystems, MA, USA) according to the manufacturer’s protocol. Paired-end raw reads for each sample (301 bp or 76 bp) were trimmed using Trimmomatic (v0.39, with the following parameters: quality 15, min_length 150, and crop 300) [7] and assembled using SPAdes (version 3.9.0, with the --careful option) [8]. Default parameters were used except where noted otherwise.

Two tulip (*Tulipa* sp.) samples were used for HTS analysis, which resulted in 3,988,106 and 6,966,540 total reads and 1,859 and 197 assembled contigs longer than 200 bp, respectively. A BLASTn analysis (https://blast.ncbi.nlm.nih.gov/Blast.cgi) revealed that 13 contigs showed similarity to MLBVV sequences: seven from RNA1, one from RNA2, four from RNA3, and one from RNA4. Nucleotide sequences similar to sequences from tulip streak virus [9, 10], tulip breaking virus, olive mild mosaic virus, and satellite tobacco necrosis virus were also found among the HTS contigs, but these were not the focus of the present study.

There were some gaps between contigs in the sequence of RNA1. Therefore, we designed TMMMV-specific primers based on the contig sequences, and the corresponding DNA fragments obtained by RT-PCR were then cloned into pGEM-T Easy Vector. The sequences were reconfirmed, excluding the terminal region of the genome. Next, we used a 5′ rapid amplification of cDNA ends (RACE) system (Invitrogen, CA, USA) to analyze the terminal genome segments. For that purpose, we designed TMMMV-specific primers based on the HTS contig sequences (Supplementary Table S1). To analyze the 3′ ends, we used a 5′ RACE system targeting the complementary RNA strand of the viral replication intermediates. The resulting sequences were assembled using ATGC version 9.0.1 (Genetyx, Tokyo, Japan), and nucleotide sequence identity values were calculated using GENETYX-MAC version 22.0.5 (Genetyx).

Complete nucleotide sequences were obtained for all four genome segments (RNA1 to RNA4) of TMMMV (Fig. 1B). The RNA1 sequence (7758 nt, 33.7% GC content) contains two open reading frames (ORFs) and 5′, 3′, and intergenic untranslated regions (UTRs) comprising 104, 88, and 132 nt, respectively. ORF1 was predicted to encode a 262-kDa RNA-dependent RNA polymerase (RdRp) protein, and an RdRp motif (Mononeg_RNA_pol_cat, InterPro IPR014023) was identified, using InterPro (https://www.ebi.ac.uk/interpro/), within the central region of ORF1 (nt positions 638-804). ORF2 encodes an unknown 23-kDa protein. RNA2 (1789 nt, 38.4% GC content) contains a single ORF (ORF4) and 5′ and 3′ UTRs comprising 322 and 15 nt, respectively. ORF4 was predicted to encode a 54-kDa movement protein (MP), including an MP motif (30K_MP_core, InterPro IPR041344) in the central region of ORF4 (positions 139-260). Although there is a predicted ORF3 (10K protein) within the viral sense strand of MLBVV (accession number AF525934), no corresponding ORF was found in the TMMMV genome. RNA3 (1514 nt, 38.4% GC content) contains a single ORF (ORF5) and 5′ and 3′ UTRs comprising 116 and 87 nt, respectively. ORF5 was predicted to encode a 48-kDa CP, and a nucleocapsid motif (Nucleocap_ssRNA, InterPro IPR021310) was found in the C-terminal region of ORF5 (nt positions 231-402). RNA4 (1432 nt, 36.9% GC content) contains a single ORF (ORF7), and 5′ and 3′ UTRs comprising 379 and 102 nt, respectively. ORF7 encodes an unknown 38-kDa protein. Although there is a predicted ORF6 (10.6K protein) within the viral complementary strand of the MLBVV RNA (accession number AF525936), no corresponding ORF was found in the TMMMV genome.

In the 5′- and 3′-terminal regions of the TMMMV genome segments, regions consisting of 1-11 nt from the ends were highly conserved in RNA1 to RNA4 (Fig. 1C). A sequence alignment of each RNA segment with those of other ophioviruses (MLBVV, lettuce ring necrosis virus, and pepper chlorosis associated virus) whose terminal sequences have been determined showed that the 5 nt at the 5′ terminus were completely conserved in all segments. Furthermore, the 10 nt at the 5′ terminus of RNA3 were completely conserved among these viruses (Fig. 1D). The 3′-terminal region was not as conserved as the 5′-terminal region, but stretches of 1-4 and 8-9 nt at the 3′ terminus were completely conserved in all segments. The 3′ terminus is U-rich, in contrast to the A-rich 5′ terminus. Furthermore, the 5′- and 3′-terminal sequences of TMMMV were found to be complementary and therefore were predicted to form duplexes (Fig. 1E). In ophioviruses, each genomic segment is predicted to form a panhandle structure in which both ends of the RNA are joined to each other [11]. This is thought to be due to the complementary binding of genomic RNA ends, and complementary sequences were also observed in TMMMV.

We constructed molecular phylogenetic trees using predicted RdRp and CP amino acid sequences and found that TMMMV clustered with MLBVV (Supplementary Fig. S1). We also found that TMMMV shared the highest sequence similarity with MLBVV in both the full-length genome and in the ORFs, whereas the CP amino acid sequence of TMMMV shared less than 85% identity with that of any other ophiovirus (Table 1). TMMMV is closely related to MLBVV, but according to the criteria for species demarcation [12], TMMMV should be considered a member of an independent species in the genus *Ophiovirus*.

**Table 1:** Sequence identity levels (%) between tulip mild mottle mosaic virus and other ophioviruses

Virus name	Nucleotide identity				Amino acid identity					Accession No.			
	RNA1	RNA2	RNA3	RNA4	ORF1	ORF2	ORF4	ORF5	ORF7	RNA1	RNA2	RNA3	RNA4
Citrus psorosis virus	50.2	44.7	47.0	ND	33.8	15.4	23.4	30.4	ND	AY654892	AY654893	AY654894	ND
Freesia sneak virus	62.6	54.7	56.1	48.1	61.6	33.0	48.1	49.7	36.5	OR539241	OR539238	OR539239	OR539240
Lettuce ring necrosis virus	62.0	54.0	56.2	49.0	61.8	29.3	45.5	52.3	39.3	AY535016	AY535017	AY535018	AY535019
Mirafiori lettuce big-vein virus	74.7	75.8	74.7	70.3	86.5	48.1	83.4	83.1	81.4	AF525933	AF525934	AF525935	AF525936
Pepper chlorosis associated virus	61.3	54.3	56.4	ND	63.2	30.6	49.6	53.1	ND	LC719619	LC719620	LC719621	ND
Ranunculus white mottle virus	61.9	55.3	56.6	47.5	62.3	34.7	50.3	54.5	34.6	OL472195	OL472208	OL472197	OL472198
Blueberry mosaic associated virus	51.2	44.3	48.0	ND	63.2	30.6	19.7	29.3	ND	KJ704366	KJ704367	KJ704368	ND

ND: No data is available

## Supplementary Information

Below is the link to the electronic supplementary material.

Supplementary Fig. S1 Unrooted neighbor-joining phylogenetic tree of TMMMV and other ophioviruses, generated using MEGA11 [13] based on the RdRp (A) and CP (B) amino acid sequences. Numbers on the branches are bootstrap values (%) obtained from 1000 replicates, and only bootstrap values higher than 60% are shown (PDF 3170 KB)

Supplementary file2 (TXT 14 KB)

## Data Availability

The tulip mild mottle mosaic virus genome sequence has been deposited in the DDBJ/ENA/GenBank database under accession numbers LC822835–8. The MiSeq data set was deposited in the NCBI Sequence Read Archive under run accession numbers DRR576617 and DRR576618.
