# Supplementary material for: First complete genome sequence of tulip mild mottle mosaic virus (Ophiovirus tulipae)
Source: Arch Virol. 2025 Jan 17;170(2):39. doi: 10.1007/s00705-025-06224-6 (PMC11742326; doi:10.1007/s00705-025-06224-6)
Supplement: Supplementary file 1 — Supplementary Fig. S1 Unrooted neighbor-joining phylogenetic tree of TMMMV and other ophioviruses, generated using MEGA11 [13] based on the RdRp (A) and CP (B) amino acid sequences. Numbers on the branches are bootstrap values (%) obtained from 1000 replicates, and only bootstrap values higher than 60% are shown (PDF 3170 KB) [file 705_2025_6224_MOESM1_ESM.pdf]

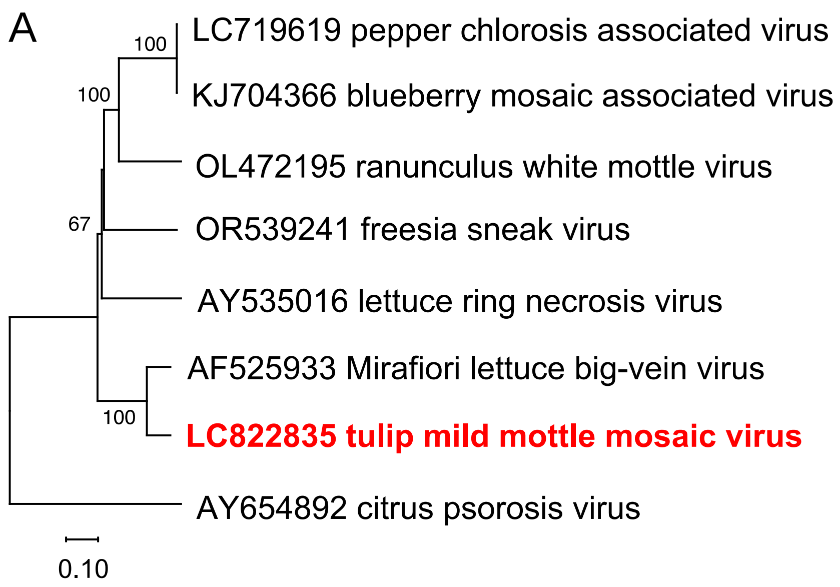

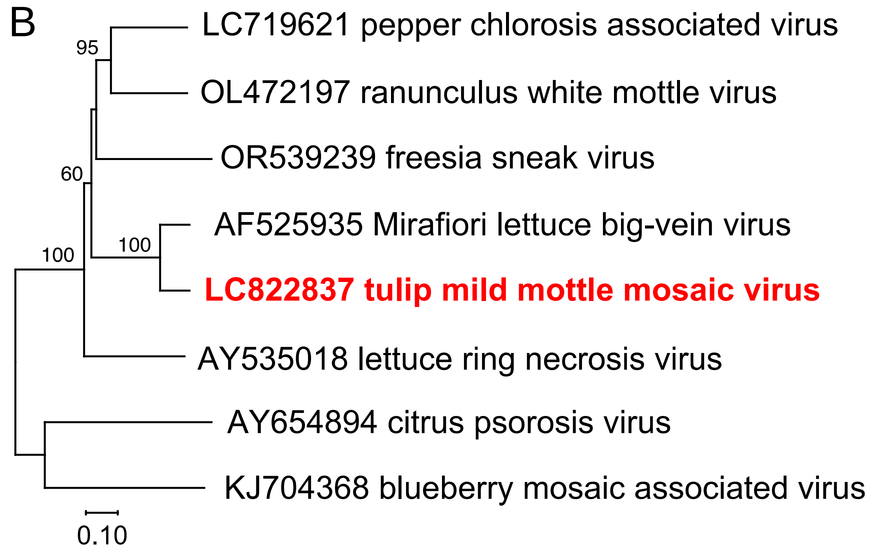

Neriya et al., Fig. S1

Table S1 Primers used in this study

Primer Name	Sequence (5' to 3')
**Amplification for RNA1 fragment**	
TMMMV3’F_RNA1	GATWMTTTTTTWMAARWAWAA
TMMMV2100R_RNA1	GATTATCTCTATTTGTTCTTTG
TMMMV2091F_RNA1	AAATAYRYACAAAGRACAAAYAGARA
TMMMV3128R_RNA1	CTTATTCCTAATTGCATAAACATATC
TMMMV3108F_RNA1	TGGGGAATACAGTCMCARATGMTAGAWGA
TMMMV4151R_RNA1	GATGAWARATCYCCTGTYTTAGGAT
TMMMV4148F_RNA1	GCGAAACCATAAGCTAAAAGTGAAG
TMMMV6089R_RNA1	CTTATCAATATAAGCCTTTTGCGCTG
TMMMV6074F_RNA1	ATCWGMWGTTTAYCCYAGCGCAAAA
TMMMV7165R_RNA1	ATYATCATTATYCTRTTMATGTATARYTT
TMMMV7131F_RNA1	GTTGGGAATAGCAAGATTAG
TMMMV7609R_RNA1	CCAWGCATCAWARASYTCTTC
**Amplification for RNA2 fragment**	
TMMMV393F_RNA2	ARWTCAAAYATTACTTTCCC
TMMMV762R_RNA2	TGAAGGAATCACAAAATAAC
TMMMV660F_RNA2	AATGAYYRGRRGMSCCAGTRTC
TMMMV1081R_RNA2	GTCCCWGTGMRWGRMAGAGCTT
TMMMV1044F_RNA2	GATTGAGACTGGAATGAAGTC
TMMMV1482R_RNA2	CCTTCTACATCCAGAGAGACTTC
**Amplification for RNA4 fragment**	
TMMMV2F_RNA4	TGATAWTTTTTTATWAAAAWTAT
TMMMV1210R_RNA4	ACYTGARRGAAATMATCAAYAT
TMMMV410F_RNA4	AAKTGGYMTCSWRGGGTAAAAG
TMMMV1310R_RNA4	GAYCCAATAWTMWCMMGKGCT
**Cloning and sequence for RNA1 fragment**	
TMM1F1	TTATTCATCACCCCAATCAC
TMM1R1	CATCAAAGAAAAAGATGATACTCAG
TMM1F2	GTATAACCCAATTGGTCAGTAAAG
TMM1R2a	CGTAGAAACACCAGCGAG
TMM1F2b	GAGTATCCATGTTTGATGATTTC
TMM1R2	GAGTTGATGGGATGTTTGAAC
TMM1F3	TCCTTTAGGACACACAACATG
TMM1F3b3	GATAAGCTGTATCCAGAAATTAAGAC
TMM1R3	GGCTTAGATCACTTTTTAGACAAAG
TMM1R3a3	AAAACTCCTTTCCAAAGATTCTTA
TMM1F4	ATCATATGTCCAAGATAATTTCCTC
TMM1R4a	TTTCATTAGACAAAATAGGAACTCC
TMM1F4b	GACATTCCACTTTTCCGGATA
TMM1R4	ATGGACTACAAAAGAGATGTGC
**Determine the 5'-end of RNA1**	
TMM1-5ter-GSP1	GTCTATTTTGAATCATA
TMM1-5ter-GSP2	GTTCGGGAAAATTTGGAAATCATTAG

**Determine the 3'-end of RNA1**	
TMM1-3ter-GSP1	ATATAAAATGAATCAATAC
TMM1-3ter-GSP2	AAAGGTGGCTGCAGCTCCTCTTCG
TMM1-3ter-GSP3	GCTGCAGCTCCTCTTCGAAAATTAAC
**Determine the 5'-end of RNA2**	
TMM2-5ter-GSP1	GAAAACTCTGGAAGATGGC
TMM2-5ter-GSP2	CTGTTCTCTGTAAAGGTGGG
**Determine the 3'-end of RNA2**	
TMM2-3ter-GSP1	ATTATCTTCAACCTGT
TMM2-3ter-GSP2	AGAGGCAACTATTGAGGCCATAGTTC
TMM2-3ter-GSP3	TGCAACCTGAGTTTCACTGGAGAAAC
**Determine the 5'-end of RNA3**	
TMM3-5ter-GSP1	TTGGAAAAAGCTAAAAA
TMM3-5ter-GSP2	CATCCACTGTCACCGGAAAGATCATC
**Determine the 3'-end of RNA3**	
TMM3-3ter-GSP1	TCCAGTTTTAACTTTTT
TMM3-3ter-GSP2	GAACAGTCCCATCTTTGCAGGAACAGC
**Determine the 5'-end of RNA4**	
TMM4-5ter-GSP1	CATTGCTCAATAATTC
TMM4-5ter-GSP2	GGCATTGATGCACTAGACGATCATG
**Determine the 3'-end of RNA4**	
TMM4-3ter-GSP1	GTTGATGATTTCTTTC
TMM4-3ter-GSP2	AATGAACCCATGGGAATTATTGAGC
TMM4-3ter-GSP3	AGCAATGAAGGTCTGAGGCAGTTTTC
